# An integrated *in vitro* approach on the enzymatic and antioxidant mechanisms of four commercially available essential oils (*Copaifera officinalis, Gaultheria fragrantissima, Helichrysum italicum,* and *Syzygium aromaticum*) traditionally used topically for their anti-inflammatory effects

**DOI:** 10.3389/fphar.2023.1310439

**Published:** 2024-01-29

**Authors:** Pilar Cebollada, Nelson G. M. Gomes, Paula B. Andrade, Víctor López

**Affiliations:** ^1^ Department of Pharmacy, Faculty of Health Sciences, Universidad San Jorge, Zaragoza, Spain; ^2^ REQUIMTE/LAQV, Laboratório de Farmacognosia, Departamento de Química, Faculdade de Farmácia, Universidade do Porto, Porto, Portugal; ^3^ Instituto Agroalimentario de Aragón-IA2, CITA-Universidad de Zaragoza, Zaragoza, Spain

**Keywords:** caryophyllene, eugenol, methyl salicylate, pinene, *Syzygium aromaticum*

## Abstract

**Introduction:** Despite the increasing number of essential oils being reported on their potential therapeutic effects, some remain relatively unknown on their biological properties. That is the case of the essential oils obtained from copaiba (*Copaifera officinalis* L.), wintergreen (*Gaultheria fragrantissima* Wall.), everlasting (*Helichrysum italicum* (Roth) G.Don) and clove (*Syzygium aromaticum* (L.) Merr. & L.M.Perry), commonly labelled as being useful on the amelioration of conditions with an inflammatory background.

**Methods:** To further broaden the current knowledge on the four essential oils, commercially available samples were approached on their effects upon a series of mediators that are involved on the inflammatory and oxidative response, both through in vitro cell-free and cell-based assays (5-lipoxygenase activity, lipid peroxidation, free radical and nitric oxide radical scavenging properties or tyrosinase inhibition).

**Results:** The four oils proved to be active at some of the concentrations tested in most of the performed assays. Significant differences were found between the essential oils, *S. aromaticum* proving to tbe the most active, followed by *G. fragrantissima* against 5-lipoxygenase (5-LOX) and linoleic acid peroxidation, proving their potential use as antioxidants and anti-inflammatory agents. In fact, the IC_50_ value of *S. aromaticum* in the 5-LOX assay was 62.30 μg mL^−1^. Besides *S. aromaticum* efficiently scavenged superoxide radicals generated by xanthine/xanthine oxidase, displaying an IC_50_ value of 135.26 μg mL^−1^. The essential oil obtained from *H. italicum* exhibited a significant decrease in the nitric oxide levels on BV-2 cells, showing its potential as a cytoprotective agent against toxic damage. Copaiba oil ranked first as the most potent tyrosinase inhibitor, exhibiting an IC_50_ 98.22 μg mL^−1^.

**Conclusion:** More studies are needed to describe the essential oils properties, but these results confirm the potential of these essential oils as anti-inflammatory and antioxidant agents.

## 1 Introduction

Essential oils can be portrayed as a mixture of natural volatile compounds, specifically low molecular weight molecules that include terpenes, alcohols, aldehydes, and ketones, being oftentimes responsible both for the bioactive properties and the aromatic fragrance. Such mixtures can be characterized by the occurrence of 20–60 compounds, with complex quantitative profiles, but with two or three compounds frequently constituting most of the total content. A detailed chemical profiling is required as while the remaining components are usually found in trace amounts, they can also modulate the biological activity of the plant-derived product. In fact, essential oils encompass constituents that exhibit nearly the complete spectrum of chemical functionalities, being usually applied topically, orally or through inhalation (Bakkali et al., 2008; Koyama and Heinbockel, 2020).

Several constituents occurring in essential oils have a wide range of chemo-ecological roles on plants, that range from insecticidal and antimicrobial effects to properties contributing to reduce the effects of abiotic stress. Oftentimes, such biological properties, displayed by the complex compounds in these products, have a translation to the development of medicinal products, cosmetics, preservatives, and perfumery products (Abelan et al., 2022). Essential oils have been playing a pivotal role in Complementary Medicine, namely in Aromatherapy, being progressively becoming popular in Western societies mainly due to the usefulness in the amelioration of pain, inflammation, sleep disorders and anxiety (Sattayakhom et al., 2023).

Specific essential oils have been continuously validated on their anti-inflammatory effects, complementing the therapeutic action of blockbuster non-steroidal anti-inflammatory drugs (NSAIDs) and steroidal anti-inflammatory drugs (SAIDs) (Bacchi et al., 2012). There is an increasing number of reports on essential oils that encompass direct anti-inflammatory effects, impacting signaling cascades, levels of cytokines, regulatory transcription factors, and the expression of pro-inflammatory genes, but also indirect effects through the neutralization of reactive nitrogen species (RNS) and reactive oxygen species (ROS) that play a pivotal role in the development and amplification of inflammatory responses (Ramsey JT et al., 2020). For example, the essential oil obtained from chamomile (*Matricaria chamomilla* L.) has been traditionally used to treat inflammation and irritation symptoms (Qasem et al., 2022) while others have been used in cosmetics and in the development of commercial products such as plant-based supplements and other products, being worth to highlight those obtained from rosemary (*Rosmarinus officinalis* L.) or lavender (*Lavandula* spp.) (Neves et al., 2018; Borges et al., 2019).

Unlike the abovementioned essential oils, some species lack experimental data substantiating their use in Complementary Medicine. Such examples include the essential oils herein investigated, i.e., from clove [*Syzygium aromaticum* (L.) Merr. & L.M.Perry] [Syn. *Eugenia caryophyllus* (Spreng.) Bullock & S.G.Harrison], copaiba (*Copaifera officinalis* L.), wintergreen (*Gaultheria fragrantissima* Wall.) and everlasting [*Helichrysum italicum* (Roth) G.Don]. While these essential oils are commonly used in the pharmaceutical industry, very few reports demonstrate their mechanisms and pharmacological properties, particularly on their ability to interfere with inflammatory mediators and as possible radical scavengers that might indirectly attenuate the exacerbation of the inflammatory response. *S. aromaticum* essential oil is mainly reputed as a food preservative, but also very frequently used in toothache and on the treatment of burns and wounds, with the herbal monograph from the European Medicines Agency validating its use on the symptomatic treatment of minor inflammations in the mouth or the throat (Committee on Herbal Medicinal Products HMPC, 2011). Besides, *S. aromaticum* is traditionally used in the treatment of vomiting, nausea, liver and stomach disorders, while also being used to treat viral, parasitic and bacterial infections (Batiha et al., 2020). Nearly all parts of *C. officinalis* are labelled as having anti-inflammatory and contraceptive effects by the native people from the Brazilian Amazon, the topical application of the essential oil being particularly effective in skin wound healing and used in massages to relief headache (da Trindade et al., 2018). Due to its high content in methyl salicylate, the anti-inflammatory effects of *G. fragrantissima* essential oil is anticipated (Mukhopadhyay et al., 2016). In fact, *Gaultheria* species have garnered growing interest within the scientific community due to the potential antioxidant activity, and therefore, their potential associated health benefits (Pandey et al., 2017). Ethnomedicinal records indicate that *H. italicum* oil soothes inflammation, simultaneously reducing pain with its built-in analgesic properties being also used in cosmetic products (Antunes Viegas et al., 2014a). Despite their wide and popular use, further experimental data is required to elucidate the mechanisms underlying the anti-inflammatory effects of the essential oils from *C. officinalis*, *G. fragrantissima*, *H. italicum* and *S. aromaticum*, which prompted us to carry out the current work. Furthermore, annotation of the anti-inflammatory effects should be accompanied by a standardized chemical profile, as the content in bioactives is dependent on the chemotype, location, season and maturation stage of the plant source.

## 2 Materials and methods

### 2.1 Reagents and chemicals

Suppliers of each reagent are indicated on the respective section. In general, enzymes, substrates, free radicals and IFN-γ were acquired from Sigma-Aldrich (St. Louis, MO, United States). Cells were acquired from the American Type Culture Collection (ATCC^®^) and cell culture reagents from GIBCO, Invitrogen™ (Gran Island, NY, United States). Spectrophotometric determinations were performed in a Multiskan GO plate reader (Thermo Fisher Scientific; Waltham, MA, United States).

### 2.2 Identification, source and chemical characterization of the essential oils

**FIGURE 1:**
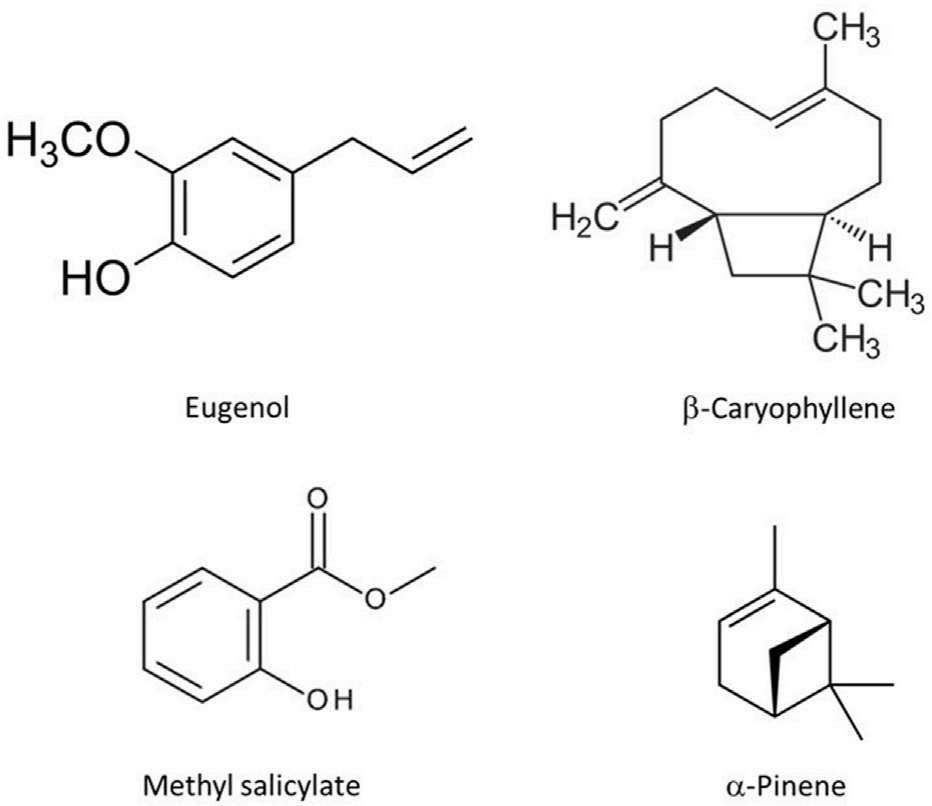
Structures of the major components of each essential oil.

Description on the four essential oils herein investigated is presented in Table 1, including the geographical location where the plant material was sourced, the plant part, as well as their chemical profiles. Essential oils were provided by Pranarôm International -Inula group (Ghislenghien, Belgium). Their chemical composition, specifically the qualitative profile in volatiles was characterized by GC-FID and provided by Pranarôm (Supplementary Figure S1) (Supplementary Table S1). More than 100 constituents have been identified on the essential oils obtained *C. officinalis* and *H. italicum*, while *S. aromaticum* essential oil is mainly characterized by the occurrence of eugenol. It is also worth to highlight that methyl salicylate encompasses more than 99% of the constituents occurring on the essential oil obtained from *G. fragrantissima*. (Table 1) (Figure 1).

**TABLE 1:** Essential oils basic information such as scientific name, batches used, country of recollection, part of the plant and main compounds.

Scientific name	Batch	Country	Part of the plant	Main compounds
*Syzygium aromaticum* (L.) Merr. & L.M.Perry	OF30342	Madagascar	Flower buds	78.19% Eugenol
				13.56% Eugenyl acetate
				4.97% E-Caryophyllene
*Copaifera officinalis* (L.)	OF38018	Brazil	Oleoresin	55.89% E-Caryophyllene
				7.51% α-Humulene
				5.91% Germacrene D + Compound Mw = 206
				4.85% α-Copaene
				3.34% δ-Cadinene
				3.30% α-*trans*-Bergamotene
*Helichrysum italicum* (Roth) G.Don	OF46599	Bosnia and Herzegovina	Flowering tops	22.44% α-Pinene + α-Thujene
				13%–21% γ-Curcumene
				7.09% β-Selinene
				6.11% Neryl acetate
				5.22% E-Caryophyllene
				4.90% Italidione I
				3.87% α-Selinene
				3.34% Italicene
				2.82% α-Curcumene
				2.22% Italidione II
				1.25% Italidione III
*Gaultheria fragrantissima* Wall	OF47135	Nepal	Leaves	99.58% Methyl salicylate

### 2.3 Inhibition of 5-lipoxygenase

5-Lipoxygenase (5-LOX) is the enzyme that participates on the conversion of arachidonic acid into leukotrienes, its inhibition being assessed by following the oxidation of linoleic acid to 13-hydroperoxylinoleic acid, according to a previously described procedure (Kachmar et al., 2019). Stock solutions of the samples were prepared in methanol 2.5%, absence of a possible interference with the assay read outs being confirmed. Samples were then dissolved in a Na_2_HPO_4_·2H_2_O (pH = 9) buffer. A minimum of six concentrations were tested, at concentrations ranging from 7.8125 to 250 μg mL^−1^. The reaction mixture was prepared in Corning^®^ 96-well clear flat bottom UV-transparent microplates as follows: 180 μL of buffer, following the addition of 20 μL of sample solution and 20 μL (100 U) of soybean 5-LOX (*Glycine max*). After 5 min of incubation at room temperature, 20 µL of a linoleic acid solution, at 4.18 mM in ethanol, were added to the mixture. The absorbance was measured at 234 nm. Three independent assays were performed in triplicate. Quercetin, tested under the same conditions, was used as a positive control at concentrations ranging from 20 to 0.625 μg mL^−1^.

### 2.4 Inhibition of lipid peroxidation

Peroxidation of linoleic acid occurs due to the abstraction of a hydrogen proton from an unsaturated lipid and involves the generation and propagation of lipid radicals, the consumption of oxygen and the rearrangement of double bonds of polyunsaturated fatty acids. The rearrangement of the double bonds results in the formation of conjugated dienes (lipid hydroperoxides), which are characterized by an intense absorption at 233 nm (Ferreres et al., 2012; Ayala et al., 2014). Essential oils were prepared in Tris-HCl buffer (100 mM, pH 7.5), six concentrations of each essential oil being prepared. 50 μL of the sample solutions were mixed with 150 µL of buffer and 250 µL of linoleic acid (20 mM in ethanol). 50 μL of a Fe SO_4_∙7H_2_O 4 mM solution in ultrapure water and 50 µL of a 5 mM ascorbic acid solution in buffer were also added to the reaction mixture. The reaction mixture was incubated during 60 min at 37°. After incubation 1.5 mL of ethanol-ether (3:1 v/v) were added to stop the reaction and the whole mixture was vortexed. Absorbance was read at 233 nm (Ferreres et al., 2012). Butylated hydroxytoluene (BHT), tested under the same conditions, was used as a positive control. Three independent experiments were performed for each essential oil.

### 2.5 Determination of NO levels in BV2 microglial cells

#### 2.5.1 Cell culture

BV-2 cells (Microglial cell *Mus musculus* (Mouse) C57BL/6) were maintained in Dulbecco’s Modified Eagle Medium (DMEM), supplemented with 10% heat-inactivated foetal bovine serum (FBS) (GIBCO, Invitrogen Grand Island, NY, United States LOT:2436448) and 1% penicillin/streptomycin (Pen Strep GIBCO, Invitrogen Grand Island, NY, United States) (10000 U mL^−1^). Cells were kept in an incubator (Toreuse model 2,428 incubator Saint Louis, MO, United States) at 37°C, in a humidified atmosphere of 5% CO_2_ (Gomes et al., 2019). Essential oils were dissolved in DMEM and filtrated through a 0.22 µm filter (TRP Spritzen-/syringe-filter 20190479).

#### 2.5.2 Interference with the mitochondrial performance of BV-2 cells

To proceed with the cell assays, it was compulsory to determine a range of working concentrations, ensuring that the effects on NO levels did not outcome from a cytotoxic effect. For that purpose, the MTT [3-(4,5-dimethylthiazolyl-2)-2,5-diphenyltetrazolium bromide] assay was performed to investigate the impact on the mitochondrial viability (Gomes et al., 2019).

BV-2 cells were plated in 96-well plates at a cell density of 40,000 cells well^−1^. After 24 h of incubation at 37°C, in a humidified atmosphere of 5% CO_2_, the medium was replaced by different concentrations of the extract, cells being cultured in that medium for another 24 h. 100 μL of MTT (0.5 mg mL^−1^) were added to each well, cells being incubated at 37°C for 90 min, with the resulting formazan crystals being later dispersed in 200 μL of DMSO:isopropanol (3:1) and quantified spectrophotometrically at 560 nm (Gomes et al., 2019).

#### 2.5.3 Impact upon NO levels as assessed by the Griess assay

BV-2 cells were plated in a 96-well microplate at a density of 40,000 cells well^−1^ and cultured for 24 h at 37°C, in a humidified atmosphere of 5% CO_2_ (Toreuse model 2,428 incubator Saint Louis, MO, United States). The cells were treated with the essential oils for 2 hours before being stimulated with 20 µL of IFN-γ. After being cultured for 22 h, under the same conditions, aliquots of 75 μL of supernatant from each well were transferred to a 96-well flat bottomed microtiter plate and mixed with 75 µL of Griess reagent (consisting of 0.1% *N*-(1-naphthyl) ethylenediamine dihydrochloride and 1% sulfanilamide in phosphoric acid 2%. After 10 min of incubation in the dark, absorbance was determined at 560 nm (Suksungworn et al., 2020).

### 2.6 ^●^NO scavenging activity assay

The ^●^NO scavenging activity assay is based on the generation of diazonium species from sulphanilamide reacting with NO_2_ and 1-naphthylethylenediamine leading to the production of an azo dye, suitable for measuring at 560 nm (Ferreres et al., 2017). Essential oils were prepared in a KH_2_PO_4_ 0.1 M buffer. Assayed concentrations ranged from 15.625 to 500 μg mL ^−1^.100 μL of the samples were added to the 96-well microplate, followed by the addition of 100 µL of SNP (sodium nitroprusside dihydrate) at 20 mM, and incubated for 60 min. 100 μL of the Griess reagent [0.1% *N*-(1-naphthyl) ethylenediamine dihydrochloride and 1% sulphanilamide in phosphoric acid 2%] were added to the plate and kept in the dark for 10 min before reading it at 560 nm. Three independent assays were performed in triplicate and the results were compared with those obtained for quercetin, tested under the same conditions, and used as a positive control.

### 2.7 Superoxide radical generated by xanthine/xanthine oxidase assay

O_2_^●−^ is formed due to the reduction of an oxygen molecule by a single electron, and can be formed via NADH but also via xanthine/xanthine oxidase. O_2_^●−^ forms a complex when reacting with NBT which absorbance can be measured allowing to describe the scavenging activity (Rodríguez-Chávez et al., 2015). The reaction mixture consisted of 90 μM xanthine (16 mM Na_2_CO_3_, and 22.8 μM NBT in phosphate buffer, pH 7.0). The assay was performed in a 96-well microplate, 240 μL of the reaction mixture were added in each well with 30 μL of the essential oil in buffer with 0.5% of methanol as cosolvent.

After, 30 μL xanthine oxidase were added, the plate was incubated in the dark for 7 min at 37°C. The absorbance is read at 560 nm (Plate reader fluorescence BIOTEK SYNERGY HI). Three independent assays were performed in triplicate. Both controls and blanks were taken into account. Gallic acid tested under the same conditions was used as a positive control.

### 2.8 Inhibition of tyrosinase

Tyrosinase enzyme inhibition was selected as another approach to assay the effects of the essential oils against oxidation. The assay was carried out in a 96-well microplate where the samples were mixed with 80 µL a potassium phosphate buffer (pH 6.8), 40 µL of L-DOPA and 40 µL of tyrosinase enzyme, both dissolved in the buffer. The inhibition of the enzyme was determined at 475 nm. Three experiments were performed in triplicate for each sample. Kojic acid was used as a positive control tested under the same conditions (Farràs et al., 2019).

### 2.9 Statistical analysis

Statistical analysis was performed with GraphPad Prism 10. On cellular assays, a Grubbs’ test was utilized to identify significant outliers. Normality was checked through D’Agostino-Pearson normality test. After, for data following a normal distribution, a one-way analysis of variance (ANOVA) with Dunnett as *post hoc* test was used to compare each experimental condition with the respective control (untreated cells). The level of significance between different groups in cell-free assays was determined by one-way ANOVA with Bonferroni’s post-test. Student´s t-test was found to be more appropriate to estimate the statistical significance of the results on the oxidation of the linoleic acid. The different values of IC_50_ for the essentials oils and positive controls were also calculated with GraphPad Prism 10.

## 3 Results

### 3.1 Inhibition of 5-lipoxygenase

**FIGURE 2:**
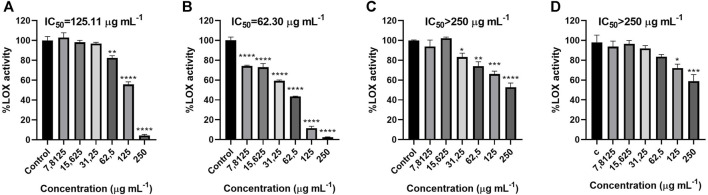
Effects of *G. fragrantissima* **(A)**, *S. aromaticum* **(B)**, *H. italicum* **(C)** and *C. officinalis* **(D)** essential oils upon 5- lipoxygenase activity levels in a cell-free assay. Results correspond to the mean ± SEM of a minimum of three independent experiments performed in triplicate (statistical significance: *p < 0.05; **p < 0.01, ***, *p* < 0.001, ****p < 0.0001) IC_50_ (quercetin) = 1.75 μg mL^−1^.

Figure 2 shows that all the essential oils significantly inhibited the 5-LOX enzyme at certain concentrations. The active concentration range for *G. fragrantissima* was from 250 to 62.5 μg mL^−1^. *H. italicum* appears to be active in the range between 250 and 31.25 μg mL^−1^ while *C. officinalis* essential oil was active only at 250 and 125 μg mL^−1^. *S. aromaticum* proved to be active in the whole range of concentrations tested, from 250–7.81 μg mL^−1^.

However, the IC_50_ value could only be determined for *G. fragrantissima and S. aromaticum.* These essential oils inhibited the enzymatic activity of 5-LOX, at their highest concentration down to 2.45% and 4.30%, respectively. Besides the oils displayed an IC_50_ value of 43.27 and 124.85 μg mL^−1^ respectively, *S. aromaticum* proving to be the most active essential oil.

### 3.2 Lipid peroxidation assay

**FIGURE 3:**
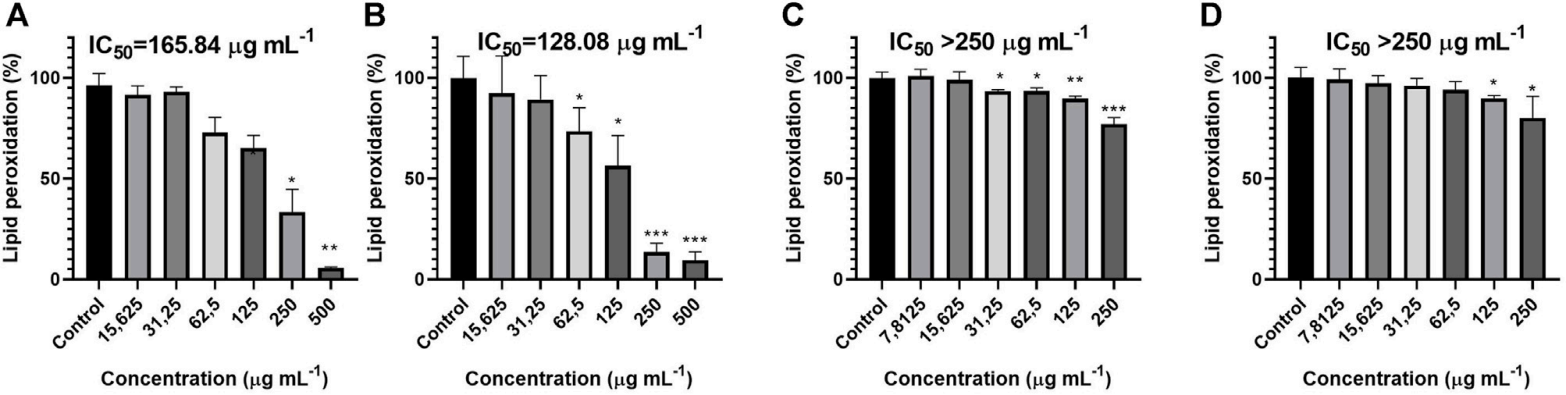
Activity of *G. fragrantissima* **(A)**, *S. aromaticum* **(B)**, *H. italicum* **(C)** and *C. officinalis* **(D)** essential oils upon lipid peroxidation levels in a cell-free assay. Results correspond to the mean ± SD of a minimum of three independent experiments (statistical significance: *p < 0.05; **p < 0.01, ***p < 0.001). IC_50_ (BHT) 871.74 µg mL−1.

As seen in Figure 3, at the highest concentrations tested, both *S. aromaticum* and *G. fragrantissima* were able to significantly decrease the lipid peroxidation down to percentages lower than 50%, 9.5%, and 5% respectively. For *C. officinalis* and *H. italicum,* the highest concentration lead to a percentage of peroxidation of the linoleic acid of 80.16% and 77.11% respectively.

The oil with a lowest IC_50_ value was found to be *S. aromaticum* with an IC_50_ of 128.08 μg mL^−1^ followed by *G. fragrantissima* with an IC_50_ of 165.84 μg mL^−1^, while the IC_50_ for *H. italicum* and *C. officinalis* were both higher than 250 μg mL^−1^. *S. aromaticum* and *G. fragrantissima* displayed an IC_50_ lower than the one calculated for the positive control.

*G. fragrantissima* and *C. officinalis* were active only in the two highest concentrations tested. For *S. aromaticum* the range of concentrations where it proved to be active was from 500 to 62.5 μg mL^−1^ while for *H. italicum* was from 250 μg mL^−1^–31.25 μg mL^−1^.

### 3.3 Cell assay in BV2 microglial cells

**FIGURE 4:**
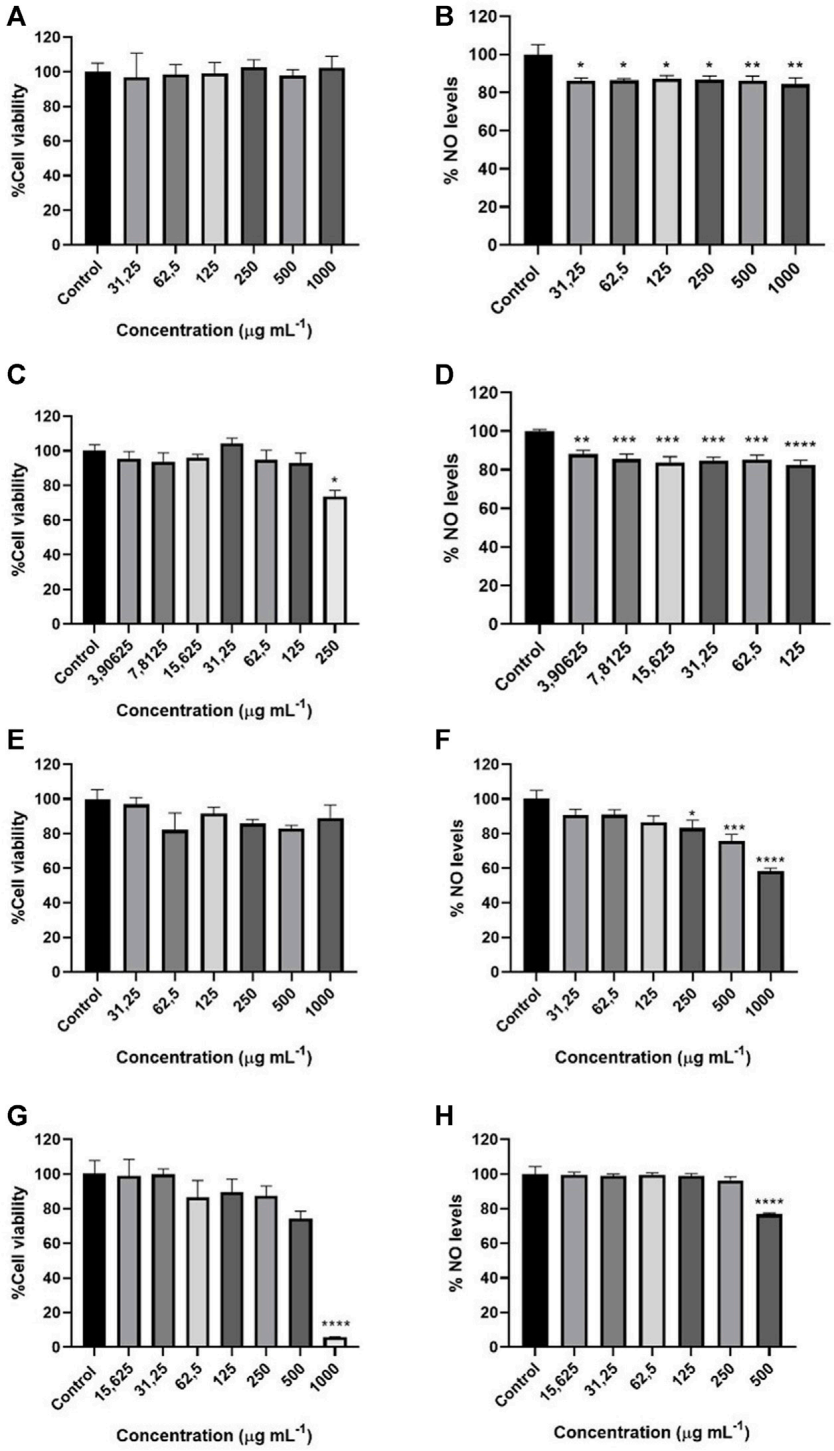
Cell viability of *G. fragrantissima* **(A)**, *S. aromaticum* **(B)**, *H. italicum* **(C)** and *C. officinalis* **(D)** essential oils. Effects of *G. fragrantissima* **(E)**, *S. aromaticum* **(F)**, *H. italicum* **(G)** and *C. officinalis* **(H)** essential oils upon ^•^NO levels in BV-2 cells assay. Results correspond to the mean ± SEM of a minimum of three independent experiments performed in triplicate (statistical significance: *p < 0.05; **p < 0.01, ***, *p* < 0.001, ****p < 0.0001).

As shown in Figure 4, the MTT assay was used to characterize the cell viability of BV2 cells in a range of concentrations from 1000 to 31.25 μg mL^−1^. *H. italicum* and *G. fragantissima* essential oils did not affect the cell viability in the tested range of concentrations. Concentrations considered safe for the cells for *S. aromaticum* were from 125 to 3.90625 μg mL^−1^ since there was a significant interference on the cellular viability at the range of concentrations from 1000 to 250 μg mL^−1^. For *C. officinalis* significant cytotoxic effects have been only recorded upon the highest concentration tested 1000 µg mL^−1^ making the select safe range for the cells from 500 to 15.625 µg mL^−1^.

For the nitrite assay studied in the range of concentrations stablished before, both *S. aromaticum* and *G. fragantissima* proved to be significantly active in the whole established range of concentrations, from 125 to 3.90625 μg mL^−1^ and 1000 to 31.25 μg mL^−1^ respectively. *C. officinalis* was found to be active only at the highest concentration tested, 500 μg mL^−1^. *H. italicum* proved to have an activity in the range of concentrations from 1000 to 250 μg mL^−1^. Even if the reduction of the nitric oxide (NO) levels was statistically significant, the IC_50_ values could not be determined since none of the oils reached a 50% reduction *H. italicum* demonstrating to be the most active essential oil on the reduction of NO levels.

### 3.4 ^●^NO scavenging activity assay

**FIGURE 5:**
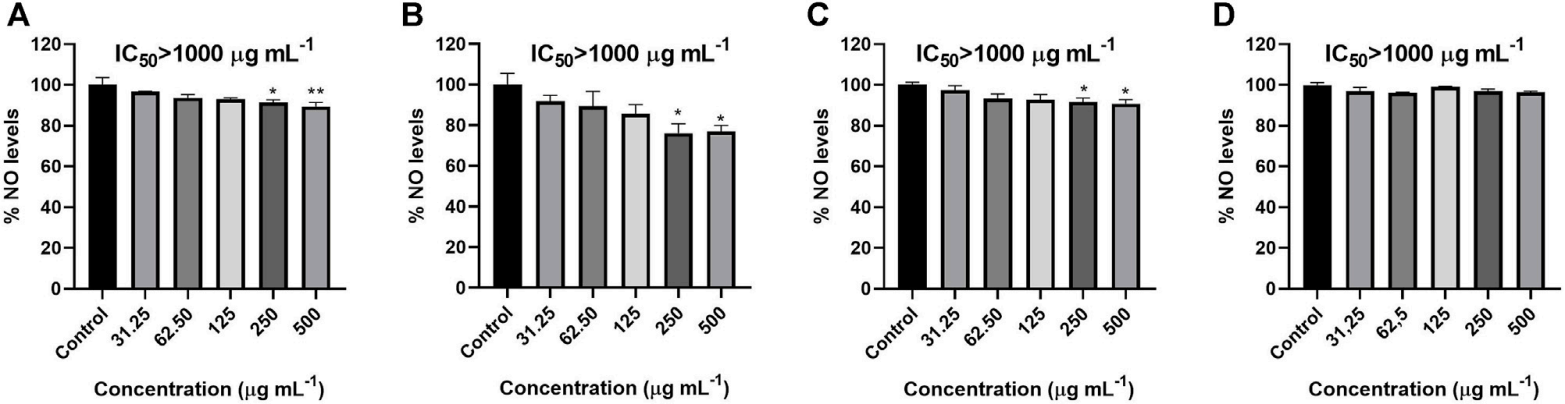
Activity of *G. fragrantissima* **(A)**, *S. aromaticum* **(B)**, *H. italicum* **(C)** and *C. officinalis* **(D)** essential oils upon ^•^NO levels in a cell-free assay. Results correspond to the mean ± SEM of a minimum of three independent experiments performed in triplicate (statistical significance: *p < 0.05; **p < 0.01) IC_50_ (quercetin) 4.89 µg mL−1.

*C. officinalis* essential oil proved to be inactive on the antiradical activity towards nitric oxide radical, but the essential oils obtained from *H. italicum, S. aromaticum* and *G. fragrantissima* significantly reduced the radical levels at 250 and 500 μg mL^−1^ despite their low activity (Figure 5). However, none of the essential oils tested in this case scavenged ^*●*^NO to a percentage lower than 50%. Therefore, the IC_50_ was higher than 500 μg mL^−1^ for all the essential oils*.* Quercetin was used as a positive control with an IC_50_ of 4.89 μg mL^−1^.

### 3.5 Superoxide radical generated by xanthine/xanthine oxidase assay

**FIGURE 6:**
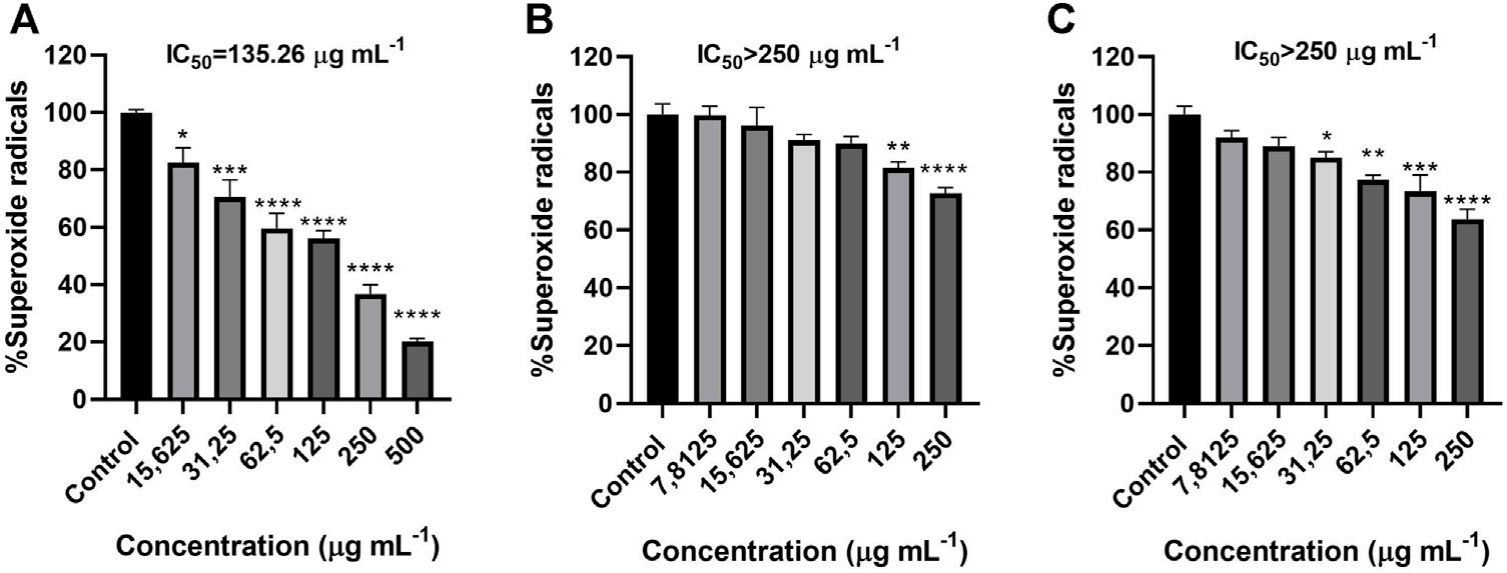
Activity of *S. aromaticum* **(A)**, *H. italicum* **(B)** and *C. officinalis* **(C)** essential oils upon superoxide levels in a cell-free assay. Results correspond to the mean ± SEM of a minimum of three independent experiments performed in triplicate (statistical significance: *p < 0.05; **p < 0.01, ***p < 0.001, ****p < 0.0001) IC_50_ (gallic acid) 0.045 μg mL^−1^.

*G. fragrantissima* essential oil was not suitable to be assayed due to the inability of the oil to solve in the buffer even with the help of cosolvents. *S. aromaticum* essential oil produced a reduction in the levels of the superoxide radicals generated by xanthine/xanthine oxidase in whole range of concentrations tested. The calculated IC_50_ for *S. aromaticum* was 135.26 μg mL^−1^ (Figure 6).

Both *H. italicum* and *C. officinalis* proved to be active in some of the concentration tested but the slight reduction in the superoxide radical levels did not allow for the IC_50_ to be determined. *H. italicum* was active from 125 μg mL^−1^ while the range displayed by *C. officinalis* was wider, from 250 to 15.625 μg mL^−1^. For both of the oils the activity at 500 μg mL^−1^ could not be determined due to solving problems (Figure 6).

### 3.6 Inhibition of tyrosinase

**FIGURE 7:**
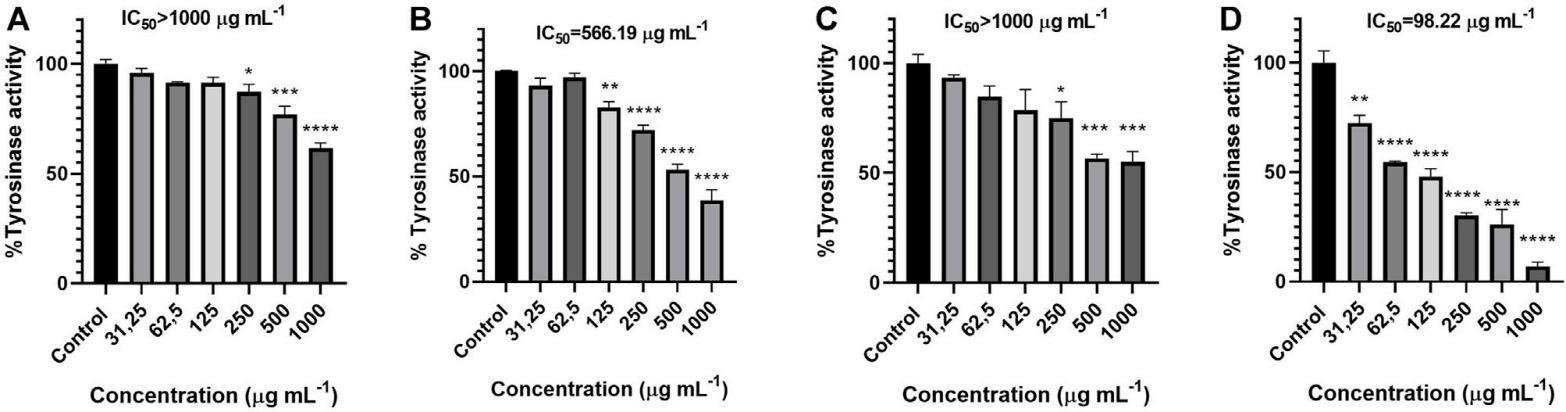
Activity of *G. fragrantissima* **(A)**, *S. aromaticum* **(B)**, *H. italicum* **(C)** and *C. officinalis* **(D)** essential oils upon tyrosinase enzyme levels in a cell-free assay. Results correspond to the mean ± SEM of a minimum of three independent experiments performed in triplicate (statistical significance: *p < 0.05; **p < 0.01, ***, *p* < 0.001, ****p < 0.0001). IC_50_ (Kojic acid) = 5.94 μg mL^−1^.

As seen in Figure 7, only *S. aromaticum* and *C. officinalis* displayed a reduction in tyrosinase activity lower than the 50% allowing for the IC_50_ values to be calculated. In particular, *C. officinalis* showed a significant reduction in all of the concentrations tested and the lowest IC_50_ value followed by *S. aromaticum.* For *G. fragrantissima* and *H. italicum* only the reduction for highest concentrations was found statistically significant.

## 4 Discussion

Wintergreen has been traditionally used as a herbal remedy in America, Canada, and India; recent reports have demonstrated its antioxidant, antibacterial and analgesic activities (Liu et al., 2013). In our work, *G. fragantissima* essential oil showed a significant activity in the inhibition of 5-LOX (Figure 2). The IC_50_ obtained for this essential oil was 124.85 μg mL^−1^, higher than the obtained for *S. aromaticum* and quercetin, used as positive control (IC_50_ = 1.75 μg mL^−1^), but still lower than those obtained from *H. italicum* and *C. officinalis* (IC_50_ > 250 μg mL^−1^). Even if the activity of the *G. fragantissima* essential oil has not been described before, Alam et al., in 2017 reported the effects of methanol and DMSO extracts obtained from other *Gaultheria* spp. (*Gaultheria trichophylla*) on the 5-LOX inhibition, obtaining similar but slightly lower activity values than the one obtained in this work (Alam et al., 2017). This seems to indicate that the essential oil has more activity as a 5-lipoxygenase inhibitor than the methanol extract and the DMSO one, perhaps due to its high content in methyl salicylate (Table 1).

The eicosanoid-metabolizing enzyme 5-LOX acts as a main actor in the process of inflammation as it underlies the synthesis of the major pro-inflammatory lipid mediators leukotrienes (LT). The inflammatory stimuli induce their biosynthesis, namely of LTA4 and further generation of LTB4 and LTC4, which boost the development of diseases such as asthma, allergic rhinitis, atherosclerosis or diabetes (Dennis and Norris, 2015).

Besides, *G. fragantissima* essential oil was found active against lipid peroxidation, decreasing significantly the percentage of linoleic acid peroxidation. Excessive oxidation of lipids alters the physical properties of cellular membranes and can cause covalent modification of proteins and nucleic acids. Peroxides resulting from this process can be classified in lipid hydroperoxides and endoperoxides which act as intermediates in the production of prostaglandins that contribute to the development of inflammation symptoms (Gaschler and Stockwell, 2017). Also, hydroxy eicosatetraenoic acids, which can be generated by lipoxygenases and nonenzymatic lipid peroxidation, can be portrayed as agonists at thromboxane receptors despite having less affinity than thromboxane A2 (Ricciotti and Fitzgerald, 2011). Therefore, the results obtained for *G. fragantissima*, dealing with the peroxidation of the linoleic acid and the 5-LOX assay indicate that the essential oil could be useful in the treatment of inflammatory related pathologies.

The results obtained in the ^●^NO scavenging activity assay were similar to those obtained from the other oils, exhibiting a significant activity at the two highest concentrations but with an IC_50_ value quite distant from the one of the positive controls (Figure 5). For the impact upon this radical produced by BV-2 cells (Figure 4), even if *G. fragantissima* essential oil is active in the whole range of concentrations tested, the highest concentration tested only reduced the levels of ^●^NO in a moderate way and the activity was lower when compared to the other oils tested in this work. Previous studies had proved a higher reduction in the NO levels on LPS-induced RAW264.7 macrophages mediated by methyl salicylate derivates obtained from *Gaultheria* species (Zhang et al., 2011).

When it comes to the tyrosinase enzyme inhibition assay, even if the reduction in the activity of the enzyme was not significant upon exposure to *G. fragantissima* essential oil, specially compared to the one exhibited by *C. officinalis*, other works have shown higher inhibition levels for other *Gaultheria* species with different composition (Fernández-Galleguillos et al., 2021).

Clove extracts and essential oils have been traditionally used as an analgesic and anti-inflammatory remedy, not coming as a surprise that the essential oil exhibited the best results in the 5-LOX assay as well as in almost all the cell free assays performed. *S. aromaticum* is the essential oil tested in this work with the lowest IC_50_ for the 5- LOX assay and the closest to the calculated IC_50_ for quercetin. Even if there are not other works studying the inhibitory activity of *S. aromaticum* essential oil on 5- LOX activity, the results obtained in this work are similar to those obtained for the clove leaf oil which showed moderate 15-lipoxygenase inhibitory activity (Wei and Shibamoto, 2010). Therefore, both works seem to indicate that the anti-inflammatory activity of *S. aromaticum* is related with the potential inhibition of the arachidonic acid metabolism. Computational analysis also showed eugenol, the major component of *S. aromaticum* essential oil, as an anti-inflammatory compound and an active 5-LOX inhibitor that could replace some NSAIDs in different diseases (das Chagas Pereira de Andrade and Mendes, 2020), being the high occurrence of eugenol a potential reason for the inhibition exhibited by the essential oil tested in this work.

For the lipid peroxidation assay *G. fragantissima* exhibits a greater reduction in the percentage of lipid peroxidation at the highest concentration than *S. aromaticum*; however, the IC_50_ for *S. aromaticum* is still lower than both the one calculated for *G. fragantissima* and the positive control (BHT). The lipid peroxidation products may lead to changes in the regulation of cellular activity and dysfunction. Indeed, some of them may activate NF-κB signaling pathways proving to be related to inflammation (Yadav and Ramana, 2013). Besides, lipid peroxidation has proved to be an important hallmark of Alzheimer’s disease cellular pathology, making specially interesting the results obtained from *S. aromaticum* (Ates et al., 2020).

Clove essential oil reduced moderately the nitric oxide levels. These results are not as promising as others obtained in different studies. Pérez-Rosés et al. (2016) described the effects of clove essential oil and eugenol on NO production by human leukocytes stimulated with LPS obtaining a high reduction in the NO levels. The effect of eugenol has also been studied in other works on the LPS-mediated NO production in RAW264.7 macrophages obtaining significant results (Li et al., 2006). That seems to indicate that *S. aromaticum* essential oil could display an higher inhibitory activity towards NO production in other cell lines.

Despite having low scavenging ability upon NO radical, the essential oil proved to have antiradical activity since it also reduced significantly the levels of O_2_^●−^, which is also involved in the generation of oxidative stress (Figure 6). The reason behind the inhibition might also be the high concentration of eugenol, has it proved in other works to scavenge the radical generated by this system (Reddy and Lokesh, 1994). Besides, the high activity of the oil could be the result of a synergistic effect between the different phenolic compounds present, even at low concentrations (Radünz et al., 2019). This antiradical and antioxidant activity seems to highlight the *S. aromaticum* essential oil use as preservative in several industries, specially considering its already described antibacterial activity (Teles et al., 2021).

*H. italicum* has been traditionally used to treat health disorders such as allergies, colds, cough, skin, liver and gallbladder disorders, inflammation, infections, and sleeplessness (Antunes Viegas et al., 2014b). There is compelling evidence that the therapeutic efficacy of this oil changes with the natural variability of the composition. In the case of the essential oil studied in this work, more than 100 constituents have been identified, being α-pinene the component found in a higher concentration (22.44%). The essential oil shows a moderate inhibition of the 5-LOX enzyme, higher than the one exhibited by *C. officinali*s but lower than the ones proved by *S. aromaticum* and *G. fragantissima,* which reduced the activity at the highest concentration to 2.45% and 4.30% respectively. Other plants species such us coriander, rich in α-pinene have also been used for its analgesic and anti-inflammatory properties (Łyczko, 2023). Even if the results were not as promising as the ones obtained for the two previous oils, the outcome of this work shows that *H. italicum* might have an anti-inflammatory potential inhibiting the arachidonic acid metabolism by 5-LOX despite not acting on the inhibition of lipid peroxidation. When it comes to the antiradical assays, *H. italicum* exhibited the highest decrease in the levels of NO in BV-2 cells within all the essential oils tested in this work. NO radical plays an important role in the regulation of inflammation since it affects every step of the process. Even at low concentrations ^●^NO starts exhibiting effects such as inhibit adhesion molecule expression, cytokine and chemokine synthesis and leukocyte adhesion and transmigration. Besides, at higher concentrations the ^●^NO radical becomes proinflammatory and toxic (Guzik et al., 2003).

Copaiba essential oil is a highly versatile product utilized across multiple industries, including the cosmetic, food and wellness industries (Urasaki et al., 2020). Even if *C. officinalis* showed the least promising activity in most of the antioxidant assays performed in this work, the essential oil displays significant inhibitory effects upon tyrosinase exhibiting the lowest IC_50_ value amongst the tested samples (Figure 7). Even if there are no works studying *C. officinalis* essential oil activity upon tyrosinase, this results match those obtained for different plant extracts that had E-Caryophyllene as one of their main components (Pintatum et al., 2020). Recent works have shown that the anti-inflammatory effects of *C. officinalis* could be related to an action on the cannabinoid receptor CB2 since E-Caryophyllene, the major component of the essential oil, has proved to selectively bind this receptor, being a functional CB2 agonist (Ferro et al., 2018). This indicates that *C. officinalis* has an anti inflammatory effect not related to lipid peroxidation, 5-LOX inhibition or antioxidant effects, that being, the ones studied in this work.

In the context of increasing demand for natural products, the present study contributes to elucidating the traditional use of these essential oils as anti-inflammatory and antioxidant agents, concurrently shedding light on various mechanisms that may be implicated. Besides, the study delivers preliminary evidence on the medicinal properties of *C. officinalis, G. fragantissima, H. italicum* and *S. aromaticum* in almost all the assays performed. A selective inhibitory effect upon the enzymatic activity of 5-LOX was exhibited by all the essential oils, suggesting the occurrence of anti-inflammatory bioactives, especially in the case of *S. aromaticum* and *G. fragantissima,* which resulted in the lowest IC_50_ values. This seems to indicate that these essential oils could be useful for the treatment of diseases related to inflammation. Future studies using animal studies or alternative models would be interesting in order to confirm the experimental *in vitro* bioactivities.

## Data Availability

The raw data supporting the conclusion of this article will be made available by the authors, without undue reservation.
