# Supplementary material for: An integrated in vitro approach on the enzymatic and antioxidant mechanisms of four commercially available essential oils (Copaifera officinalis, Gaultheria fragrantissima, Helichrysum italicum, and Syzygium aromaticum) traditionally used topically for their anti-inflammatory effects
Source: Front Pharmacol. 2024 Jan 29;14:1310439. doi: 10.3389/fphar.2023.1310439 (PMC10871035; doi:10.3389/fphar.2023.1310439)
Supplement: Supplementary file 1 [file Table1.DOCX]

Supplementary Material

# Supplementary Figures and Tables

Supplementary Figure 1 (A): Chromatographic Profile *G. fragrantissima* OF47135


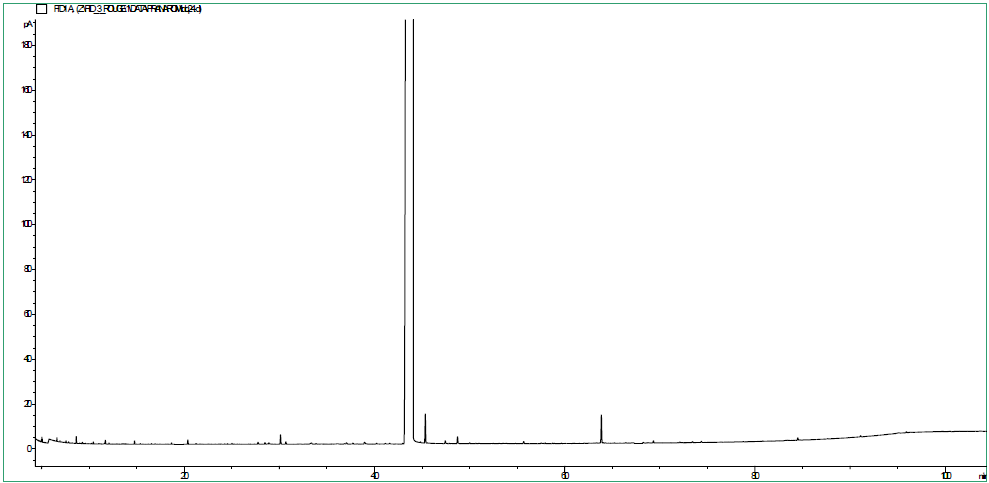


Supplementary Table 1 (A): Chromatographic Profile Results *G. fragrantissima* OF47135

| PICS | RETENTION TIME | CONSTITUENTS | % |
| --- | --- | --- | --- |
| 1 | 6.6 | Alpha-pinene | 0.01 |
| 2 | 7.5 | Camphene | 0.01 |
| 3 | 8.6 | Beta-pinene | 0.01 |
| 4 | 10.4 | Alpha-phellandrene | 0.01 |
| 5 | 11.6 | Limonene | 0.01 |
| 6 | 14.7 | p-Cymene | 0.01 |
| 7 | 18.6 | 1-hexanol | 0.01 |
| 8 | 20.4 | 3-hexen-1-ol | 0.01 |
| 9 | 30.1 | Linalool | 0.03 |
| 10 | 30.6 | 1-octanol | 0.01 |
| 11 | 33.3 | Beta-caryophyllene | 0.01 |
| 12 | 37.0 | Ethyl benzoate | 0.01 |
| 13 | 38.9 | Alpha-Terpineol | 0.01 |
| 14 | 44.0 | **Methyl salicylate** | **99.58** |
| 15 | 45.3 | Ethyl salicylate | 0.09 |
| 16 | 47.4 | Geraniol | 0.01 |
| 17 | 48.7 | Benzyl alcohol | 0.02 |
| 18 | 55.6 | Phenol | 0.01 |
| 19 | 63.8 | Eugenol | 0.10 |
| 20 | 69.3 | Cinnamyl alcohol | 0.01 |
| 21 | 74.3 | Methyl stearate | 0.01 |
| 22 | 84.5 | Phytol | 0.01 |
| **TOTAL** | | **99.99** | |

Supplementary Figure 1 (B): Chromatographic Profile *S. aromaticum* OF30342


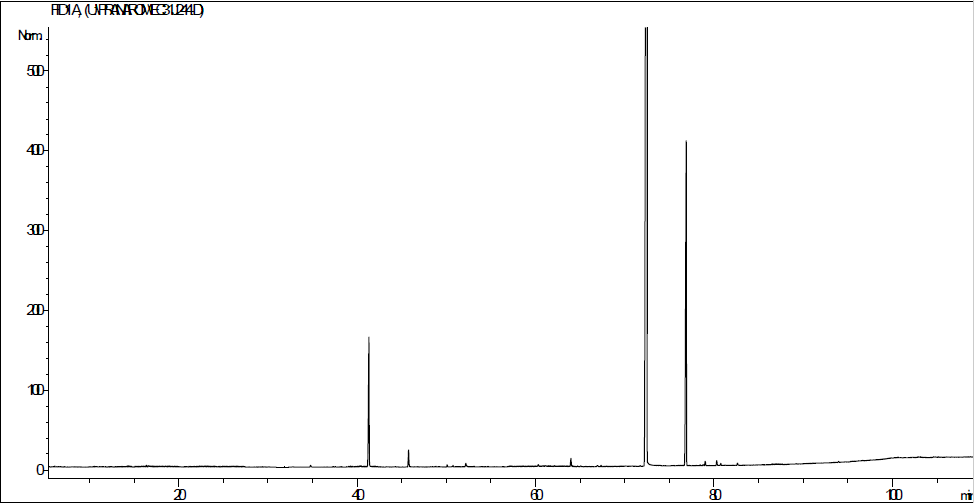


Supplementary Table 1 (B): Chromatographic Profile Results *S. aromaticum* OF30342

| PICS | RETENTION TIME | CONSTITUENTS | % |
| --- | --- | --- | --- |
| 1 | 32.7 | Furfuraldehyde | 0.03 |
| 2 | 34.8 | Alpha -Copaene | 0.07 |
| 3 | 37.5 | Linalool | 0.03 |
| 4 | 40.4 | Methyl furfural | 0.04 |
| 5 | 41.3 | Beta -Caryophyllene | **4.97** |
| 6 | 45.4 | Ethyl benzoate + zonarene | 0.01 |
| 7 | 45.8 | Alpha -Humulene | 0.63 |
| 8 | 46.8 | Gamma-muurolene | 0.02 |
| 9 | 48.2 | Sesquiterpene | 0.01 |
| 10 | 48.4 | Sesquiterpene | 0.01 |
| 11 | 48.8 | Benzyl acetate | 0.01 |
| 12 | 50.0 | Alpha -Farnesene | 0.06 |
| 13 | 50.7 | Delta-cadinene | 0.06 |
| 14 | 51.0 | Gamma-cadinene | 0.02 |
| 15 | 52.2 | Methyl salicylate | 0.19 |
| 16 | 57.1 | Trans-jasmone | 0.11 |
| 17 | 60.3 | Chavicyl acetate | 0.20 |
| 18 | 62.1 | Isocaryophyllene oxide | 0.10 |
| 19 | 63.9 | Caryophyllene oxide | 0.33 |
| 20 | 64.7 | Sesquiterpenique epoxyde | 0.03 |
| 21 | 65.0 | Methyleugenol | 0.03 |
| 22 | 66.9 | Humulene-6,7-epoxide | 0.06 |
| 23 | 67.3 | Caryophyllenol | 0.05 |
| 24 | 67.9 | Sesquiterpenol | 0.04 |
| 25 | 71.7 | Sesquiterpenol | 0.03 |
| 26 | 72.4 | **Eugenol** | **78.19** |
| 27 | 73.8 | Sesquiterpenol | 0.04 |
| 28 | 76.0 | Sesquiterpenol | 0.02 |
| 29 | 76.8 | **Eugenyl acetate** | **13.56** |
| 30 | 77.2 | Sesquiterpenol | 0.02 |
| 31 | 78.4 | Sesquiterpenol | 0.04 |
| 32 | 78.8 | Caryophylladiene isomere | 0.04 |
| 33 | 79.0 | Caryophylla-3,7-dien-6-ol | 0.16 |
| 34 | 80.3 | Chavicol | 0.18 |
| 35 | 80.8 | Diterpene mw=272 | 0.10 |
| 36 | 81.2 | Isoeugenol | 0.08 |
| 37 | 82.1 | Sesquiterpenique epoxyde | 0.09 |
| 38 | 82.6 | Sesquiterpenique epoxyde | 0.19 |
| 39 | 93.9 | Benzyl benzoate | 0.04 |
| 40 | 100.6 | Benzyl salicylate | 0.04 |
| 41 | 104.6 | Palmitic acid | 0.06 |
| **TOTAL** | | **99.99** | |

Supplementary Figure 1 (C): Chromatographic Profile *H. italicum* OF46599


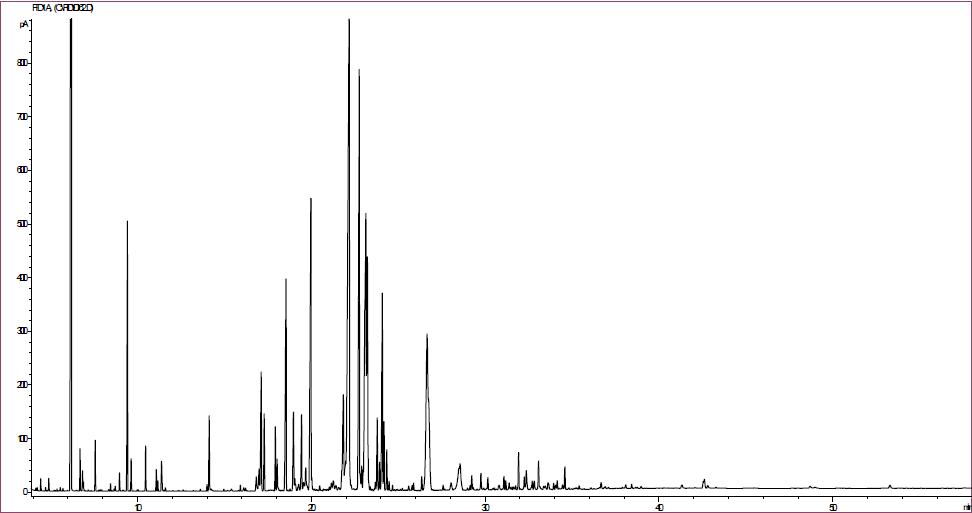


Supplementary Table 1 (C): Chromatographic Profile Results *H. italicum* OF46599

| PICS | RETENTION TIME | CONSTITUENTS | % |
| --- | --- | --- | --- |
| 1 | 4.1 | Octane | 0.02 |
| 2 | 4.2 | Acetone | 0.03 |
| 3 | 4.4 | 2-methyl-2-heptene | 0.09 |
| 4 | 4.7 | Nonane | 0.03 |
| 5 | 4.9 | Ethanol | 0.10 |
| 6 | 5.4 | Propanone isomer | 0.02 |
| 7 | 5.6 | Bornylene | 0.03 |
| 8 | 5.7 | 2-methyl-3-pentanone | 0.03 |
| 9 | 6.2 | **Alpha-pinene** + alpha-thujene | **22.44** |
| 10 | 6.7 | Alpha-fenchene | 0.34 |
| 11 | 6.8 | Camphene | 0.16 |
| 12 | 6.9 | 4-methyl-3-hexanone | 0.07 |
| 13 | 7.6 | Beta-pinene | 0.44 |
| 14 | 7.8 | Pinadiene | 0.01 |
| 15 | 7.9 | Delta2-carene | 0.01 |
| 16 | 8.3 | Compound mw=152 | 0.02 |
| 17 | 8.4 | Beta-myrcene | 0.06 |
| 18 | 8.6 | Alpha-phellandrene | 0.02 |
| 19 | 8.7 | Isobutyl 2-methylbutyrate | 0.04 |
| 20 | 9.0 | Alpha-terpinene | 0.17 |
| 21 | 9.4 | Limonene | 2.75 |
| 22 | 9.6 | 1,8-cineole | 0.30 |
| 23 | 10.0 | 2-pentyl-furane | 0.01 |
| 24 | 10.1 | Cis-beta-ocimene | 0.01 |
| 25 | 10.5 | Gamma-terpinene | 0.41 |
| 26 | 11.1 | P-cymene | 0.20 |
| 27 | 11.2 | 2-methylbutyrate de 2-methylbutyle | 0.10 |
| 28 | 11.4 | Terpinolene + Isobutyl angelate | 0.41 |
| 29 | 11.6 | Amyl isovalerate | 0.03 |
| 30 | 12.6 | Aliphatic ester | 0.01 |
| 31 | 13.6 | Aliphatic ester | 0.02 |
| 32 | 13.9 | 2-Methylbutyl angelate | 0.03 |
| 33 | 14.0 | 2-Nonanone | 0.03 |
| 34 | 14.1 | Isoamyl angelate | 0.78 |
| 35 | 14.2 | Nonanal | 0.04 |
| 36 | 15.0 | Hexyl 2-methylbutanoate | 0.02 |
| 37 | 15.4 | Methyl-p-cresol | 0.02 |
| 38 | 15.9 | Aliphatic ester | 0.07 |
| 39 | 16.0 | Hexyl angelate | 0.04 |
| 40 | 16.1 | Aliphatic ester | 0.01 |
| 41 | 16.2 | Neryl oxide | 0.03 |
| 42 | 16.8 | Ylangene | 0.21 |
| 43 | 16.9 | Cyclosativene + isoledene | 0.04 |
| 44 | 17.0 | Sesquiterpene | 0.36 |
| 45 | 17.1 | Alpha-copaene | 1.67 |
| 46 | 17.3 | Isoitalicene | 0.92 |
| 47 | 17.7 | 2,4-dimethyl-heptane-3,5-dione | 0.05 |
| 48 | 17.8 | Sesquiterpene | 0.02 |
| 49 | 17.9 | Linalool | 0.63 |
| 50 | 18.0 | Hexyl angelate isomer | 0.33 |
| 51 | 18.1 | Alpha-gurjunene | 0.05 |
| 52 | 18.6 | Italicene | 3.34 |
| 53 | 18.7 | Sesquiterpene | 0.01 |
| 54 | 18.8 | Alpha-cedrene | 0.02 |
| 55 | 18.9 | Alpha-cis-bergamotene | 1.15 |
| 56 | 19.0 | Italicene isomer | 0.13 |
| 57 | 19.1 | Italicene isomer | 0.08 |
| 58 | 19.2 | 3,5-dimethyloctane-4,6-dione | 0.47 |
| 59 | 19.3 | Cedrene isomer | 0.04 |
| 60 | 19.4 | Fenchol | 0.08 |
| 61 | 19.5 | Alpha-trans-bergamotene | 0.95 |
| 62 | 19.6 | 2-undecanone | 0.05 |
| 63 | 19.7 | Terpinene-4-ol | 0.20 |
| 64 | 19.9 | Beta-caryophyllene | 5.22 |
| 65 | 20.0 | 6,9-guaiadiene | 0.15 |
| 66 | 20.1 | Sesquiterpene | 0.02 |
| 67 | 20.2 | Aromadendrene | 0.02 |
| 68 | 20.3 | Compound mw=168 | 0.02 |
| 69 | 20.5 | Sesquiterpene | 0.06 |
| 70 | 20.7 | Beta-santalene | 0.02 |
| 71 | 20.8 | 1-nonanol | 0.01 |
| 72 | 20.9 | Germacrene a | 0.02 |
| 73 | 21.0 | Allo-aromadendrene | 0.04 |
| 74 | 21.2 | Curcumene isomer | 0.12 |
| 75 | 21.3 | E-beta-farnesene | 0.22 |
| 76 | 21.4 | Zonarene | 0.11 |
| 77 | 21.5 | Sesquiterpene | 0.04 |
| 78 | 21.6 | Sesquiterpene | 0.02 |
| 79 | 21.7 | Alpha-humulene | 0.24 |
| 80 | 21.8 | Gamma-selinene + curcumene isomer | 1.77 |
| 81 | 21.9 | Curcumene isomer | 0.26 |
| 82 | 22.0 | Gamma-muurolene | 0.33 |
| 83 | 22.1 | Gamma-curcumene | 13.21 |
| 84 | 22.2 | Borneol | 0.06 |
| 85 | 22.3 | Calarene | 0.03 |
| 86 | 22.4 | Sesquiterpene | 0.01 |
| 87 | 22.5 | Germacrene d | 0.02 |
| 88 | 22.6 | Sesquiterpene | 0.02 |
| 89 | 22.7 | Neryl acetate | 6.11 |
| 90 | 22.8 | Sesquiterpene | 0.22 |
| 91 | 22.9 | Eremophilene | 0.31 |
| 92 | 23.0 | Alpha-muurolene | 0.20 |
| 93 | 23.1 | Beta-selinene | 7.09 |
| 94 | 23.2 | Alpha-selinene | 3.87 |
| 95 | 23.3 | Beta-bisabolene | 0.07 |
| 96 | 23.4 | Cis-alpha-bisabolene | 0.04 |
| 97 | 23.5 | Sesquiterpene | 0.02 |
| 98 | 23.6 | Eudesmatriene isomer | 0.01 |
| 99 | 23.7 | E,e-alpha-farnesene | 0.09 |
| 100 | 23.8 | Delta-cadinene | 0.89 |
| 101 | 23.9 | Gamma-cadinene | 0.33 |
| 102 | 24.0 | Alpha-curcumene | 2.82 |
| 103 | 24.1 | Neryl propionate | 0.78 |
| 104 | 24.2 | Trans-alpha-bisabolene | 0.02 |
| 105 | 24.3 | Nerol | 0.45 |
| 106 | 24.5 | Cadina-1,4-diene | 0.12 |
| 107 | 24.7 | Alpha-amorphene | 0.06 |
| 108 | 24.8 | Selinadiene isomer | 0.02 |
| 109 | 24.9 | 2,4-decadienal | 0.01 |
| 110 | 25.0 | Sesquiterpene mw=202 | 0.02 |
| 111 | 25.1 | Aromatique compound mw=194 | 0.02 |
| 112 | 25.2 | Menthatrienique compound | 0.03 |
| 113 | 25.6 | Calamenene | 0.06 |
| 114 | 25.8 | Neryle isobutyrate | 0.06 |
| 115 | 25.9 | Compound mw=220 | 0.08 |
| 116 | 26.4 | Neryl 2-methylbutyrate | 0.18 |
| 117 | 26.7 | Italidione i | 4.90 |
| 118 | 26.8 | Italidione ii | 2.22 |
| 119 | 27.0 | Benzoique ester | 0.01 |
| 120 | 27.6 | Alpha-calacorene | 0.06 |
| 121 | 27.7 | Cis-jasmone | 0.01 |
| 122 | 28.0 | Italidione isomer | 0.20 |
| 123 | 28.2 | Italidione iii | 1.25 |
| 124 | 28.6 | Neryl valerate | 0.11 |
| 125 | 29.0 | Beta-calacorene | 0.03 |
| 126 | 29.1 | 2-Phenylethyl isovalerate | 0.05 |
| 127 | 29.2 | Caryophyllene oxide | 0.17 |
| 128 | 29.3 | Neryle methylvalerate | 0.03 |
| 129 | 29.5 | Sesquiterpenique epoxyde | 0.01 |
| 130 | 29.8 | Nerolidol | 0.20 |
| 131 | 29.9 | Aliphatic dione | 0.02 |
| 132 | 30.1 | Sesquiterpenique epoxyde | 0.02 |
| 133 | 30.2 | Neryl caproate | 0.14 |
| 134 | 30.4 | Cedrene oxide | 0.02 |
| 135 | 30.5 | Caryophyllenol | 0.02 |
| 136 | 30.8 | Cubenol | 0.04 |
| 137 | 30.9 | Sesquiterpenol | 0.06 |
| 138 | 31.1 | Guaiol | 0.15 |
| 139 | 31.2 | Compound mw=235 | 0.11 |
| 140 | 31.3 | 2-Phenylethyl tiglate | 0.09 |
| 141 | 31.4 | Sesquiterpenone | 0.02 |
| 142 | 31.5 | Aliphatic dione | 0.03 |
| 143 | 31.6 | 10-epi-gamma-eudesmol | 0.04 |
| 144 | 31.8 | Eudesmol isomer | 0.05 |
| 145 | 31.9 | Eudesma-5-en-11-alpha-ol | 0.44 |
| 146 | 32.0 | Sesquiterpenol | 0.01 |
| 147 | 32.3 | Sesquiterpenique epoxyde | 0.18 |
| 148 | 32.4 | Beta-bisabolol | 0.33 |
| 149 | 32.6 | Compound mw=250 | 0.02 |
| 150 | 32.7 | 7-epi-beta-eudesmol | 0.10 |
| 151 | 32.8 | Gamma-eudesmol | 0.14 |
| 152 | 33.1 | Levojuneol | 0.37 |
| 153 | 33.2 | Sesquiterpenique epoxyde | 0.04 |
| 154 | 33.3 | Aromatic compound | 0.05 |
| 155 | 33.4 | Hinesol | 0.02 |
| 156 | 33.5 | Bulnesol | 0.06 |
| 157 | 33.6 | Epi-alpha-bisabolol | 0.07 |
| 158 | 33.7 | Aromatic compound mw=208 | 0.08 |
| 159 | 33.6 | Aromatic compound mw=208 | 0.08 |
| 160 | 33.9 | Alpha-eudesmol | 0.07 |
| 161 | 34.1 | Alpha-cadinol | 0.06 |
| 162 | 34.2 | Beta-eudesmol | 0.11 |
| 163 | 34.3 | Cetonic compound | 0.02 |
| 164 | 34.5 | Farnesyl acetate | 0.07 |
| 165 | 34.6 | Eudesma-7(11)-en-4-ol | 0.28 |
| 166 | 34.8 | Capric acid | 0.02 |
| 167 | 35.1 | Cis-alpha-bisabolol | 0.02 |
| 168 | 35.2 | Compound mw=150 | 0.02 |
| 169 | 35.3 | Bergamotol isomer | 0.02 |
| 170 | 35.4 | Caryophylla-2,6-dien-5-alpha-ol | 0.05 |
| 171 | 36.1 | Sesquiterpenique epoxyde | 0.02 |
| 172 | 36.6 | Bergamotol isomer | 0.01 |
| 173 | 36.7 | Aliphatic dione | 0.10 |
| 174 | 36.8 | Aromatic compound | 0.01 |
| 175 | 36.9 | Aromatic compound | 0.04 |
| 176 | 37.1 | Bergamotol isomer | 0.02 |
| 177 | 37.9 | Aromatic compound | 0.02 |
| 178 | 38.1 | Aliphatic dione | 0.06 |
| 179 | 38.4 | Lauric acid | 0.05 |
| 180 | 38.7 | Aromatic compound | 0.02 |
| 181 | 38.8 | Aromatic compound | 0.02 |
| 182 | 39.0 | Aliphatic dione | 0.03 |
| 183 | 41.3 | Aliphatic dione | 0.04 |
| 184 | 41.4 | Aliphatic dione | 0.04 |
| 185 | 41.7 | Phytol | 0.01 |
| 186 | 42.1 | Benzyle benzoate | 0.02 |
| 187 | 42.6 | Aliphatic dione | 0.10 |
| 188 | 42.7 | Aliphatic dione | 0.16 |
| 189 | 42.8 | Aliphatic dione | 0.06 |
| 190 | 43.3 | Xanthorrhizol | 0.02 |
| 191 | 48.7 | Compound sesquiterpenic | 0.06 |
| 192 | 49.0 | Compound sesquiterpenic | 0.03 |
| 193 | 53.3 | Squalene | 0.08 |
| **Total** | | **99.98** | |

Supplementary Figure 1 (D): Chromatographic Profile *C.* *officinalis* OF38018


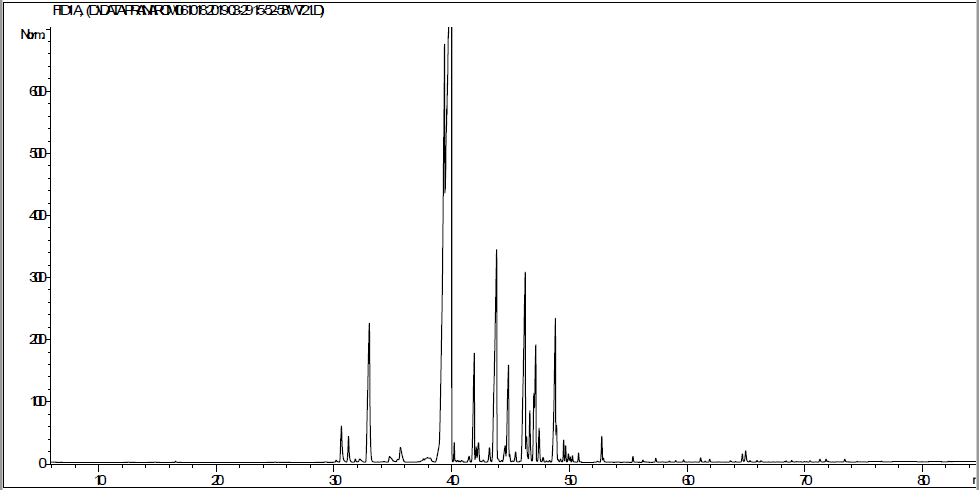


Supplementary Table 1 (D): Chromatographic Profile Results *C.* *officinalis* OF38018

| PICS | RETENTION TIME | CONSTITUENTS | % | |
| --- | --- | --- | --- | --- |
| 1 | 12.5 | Aliphatic alcohol | 0.01 | |
| 2 | 14.8 | Limonene | 0.01 | |
| 3 | 16.5 | Cis-beta-ocimene | 0.01 | |
| 4 | 24.9 | Allo-ocimene isomer | 0.01 | |
| 1 | 29.2 | Sesquiterpene | 0.01 | |
| 6 | 30.0 | Sesquiterpene | 0.01 | |
| 7 | 30.1 | Sesquiterpene | 0.04 | |
| 8 | 30.2 | Sesquiterpene | 0.01 | |
| 9 | 30.6 | Alpha-cubebene | 0.91 | |
| 10 | 31.2 | Delta-elemene | 0.54 | |
| 11 | 31.8 | Bicycloelemene | 0.06 | |
| 12 | 32.1 | Cyclosativene + ylangene | 0.13 | |
| 13 | 33.0 | **Alpha-copaene** | **4.85** | |
| 14 | 34.7 | Cyperene | 0.27 | |
| 15 | 35.4 | Bergamotene isomer | 0.07 | |
| 16 | 35.6 | Beta1-cubebene | 0.56 | |
| 17 | 37.6 | Sesquiterpene | 0.12 | |
| 18 | 37.9 | Calarene | 0.40 | |
| 19 | 39.4 | **Alpha-trans-bergamotene** | **3.30** | |
| 20 | 39.9 | **Beta-caryophyllene** | **55.89** | |
| 21 | 40.2 | Aromadendrene | 0.23 | |
| 22 | 40.3 | Sesquiterpene | 0.02 | |
| 23 | 40.5 | Himachalene isomer | 0.03 | |
| 24 | 40.8 | Cadinadiene isomer | 0.04 | |
| 25 | 41.3 | Cadina-3,5-diene | 0.02 | |
| 26 | 41.4 | Epi-beta-santalene | 0.10 | |
| 27 | 41.9 | Germacrene a | 2.29 | |
| 28 | 42.0 | Sesquiterpene | 0.26 | |
| 29 | 42.2 | Allo-aromadendrene | 0.45 | |
| 30 | 42.7 | Sesquiterpene + farnesene isomer | 0.05 | |
| 31 | 43.2 | Zonarene + sesquiterpene | 0.32 | |
| 32 | 43.8 | **Alpha-humulene** | **7.51** | |
| 33 | 43.9 | E-beta-farnesene | 0.08 | |
| 34 | 44.2 | Sesquiterpene | 0.04 | |
| 35 | 44.5 | Z-beta-farnesene | 0.43 | |
| 36 | 44.8 | Gamma-muurolene | 2.32 | |
| 37 | 44.9 | Gamma-curcumene | 0.10 | |
| 38 | 45.4 | Ledene | 0.21 | |
| 39 | 46.2 | **Germacrene d +** compound mw=206 | **5.91** | |
| 40 | 46.3 | Muurolene isomer | 0.49 | |
| 41 | 46.6 | Beta-selinene | 0.79 | |
| 42 | 46.7 | Sesquiterpene | 0.13 | |
| 43 | 46.9 | Alpha-selinene | 1.16 | |
| 44 | 47.1 | Beta-bisabolene + bicyclogermacrene | 2.34 | |
| 45 | 47.4 | Alpha-muurolene | 0.64 | |
| 46 | 47.7 | Curcumene isomer | 0.09 | |
| 47 | 48.8 | Delta-cadinene | 3.34 | |
| 48 | 48.9 | Gamma-cadinene | 0.52 | |
| 49 | 49.0 | Sesquiterpene | 0.04 | |
| 50 | 49.2 | Delta-selinene | 0.05 | |
| 51 | 49.5 | Beta-sesquiphellandrene | 0.31 | |
| 52 | 49.6 | Alpha-curcumene | 0.22 | |
| 53 | 49.9 | Cis-alpha-bisabolene | 0.12 | |
| 54 | 50.0 | Selinadiene isomer | 0.08 | |
| 55 | 50.2 | Cadina-1,4-diene | 0.10 | |
| 56 | 50.7 | Alpha-amorphene | 0.14 | |
| 57 | 52.4 | Sesquiterpene | 0.02 | |
| 58 | 52.7 | Germacrene b | 0.40 | |
| 59 | 52.9 | Calamenene | 0.06 | |
| 60 | 54.1 | E-geranyl acetone | 0.01 | |
| 61 | 55.4 | Aliphatic ester | 0.08 | |
| 62 | 56.2 | Épi-cubebol | 0.03 | |
| 63 | 57.3 | Alpha-calacorene | 0.06 | |
| 64 | 57.8 | Humulene oxide | 0.01 | |
| 65 | 58.5 | Sesquiterpene epoxide | 0.02 | |
| 66 | 58.8 | Aliphatic alcohol | 0.01 | |
| 67 | 59.0 | Cubebol | 0.02 | |
| 68 | 59.7 | Beta-calacorene + Aliphatic ester | 0.04 | |
| 69 | 60.1 | Sesquiterpene epoxide | 0.01 | |
| 70 | 60.7 | Isocaryophyllene | 0.01 | |
| 71 | 61.1 | Caryophyllene oxide | 0.07 | |
| 72 | 61.5 | Sesquiterpenol | 0.01 | |
| 73 | 61.9 | Sesquiterpenol | 0.04 | |
| 74 | 63.6 | Ledol | 0.03 | |
| 75 | 64.1 | Epoxy-6,7-humulene | 0.01 | |
| 76 | 64.7 | Caryophyllenol | 0.14 | |
| 77 | 64.9 | Juneol + épi-cubenol | 0.24 | |
| 78 | 65.3 | Cubenol | 0.03 | |
| 79 | 65.9 | Globulol | 0.03 | |
| 80 | 66.3 | Viridiflorol | 0.03 | |
| 81 | 67.3 | Sesquiterpenol | 0.01 | |
| 82 | 67.6 | Epi-gamma-eudesmol | 0.01 | |
| 83 | 67.8 | Rosifoliol | 0.01 | |
| 84 | 68.2 | Spathulenol | 0.01 | |
| 85 | 68.4 | Dihydroisocaryophyllene mw=206 | 0.02 | |
| 86 | 68.9 | Sesquiterpenol | 0.03 | |
| 87 | 69.2 | Epi-cubenol | 0.01 | |
| 88 | 69.3 | Cubenol | 0.01 | |
| 89 | 69.6 | Sesquiterpene epoxide | 0.01 | |
| 90 | 70.0 | Compound mw=206 | 0.01 | |
| 91 | 70.1 | Sesquiterpenol | 0.01 | |
| 92 | 70.5 | T-cadinol | 0.03 | |
| 93 | 71.2 | Alpha-muurolol | 0.05 | |
| 94 | 71.6 | Sesquiterpenol | 0.01 | |
| 95 | 71.8 | Delta-cadinol | 0.04 | |
| 96 | 71.9 | Sesquiterpenol | 0.02 | |
| 97 | 72.6 | Epi-alpha-bisabolol | 0.01 | |
| 98 | 72.7 | Alpha-bisabolol | 0.01 | |
| 99 | 73.0 | Sesquiterpenol | 0.01 | |
| 100 | 73.4 | Alpha-cadinol | 0.05 | |
| 101 | 74.4 | Eudesma-7-en-4-ol | 0.01 | |
| 102 | 76.5 | Sesquiterpenol | 0.01 | |
| 103 | 82.2 | Benzoic acid | 0.01 | |
| **TOTAL** | | **99.99** | |  |
